# Inflammatory myopathy occurring shortly after severe acute respiratory syndrome coronavirus 2 vaccination: two case reports

**DOI:** 10.1186/s13256-022-03266-1

**Published:** 2022-01-30

**Authors:** Kritchai Vutipongsatorn, Anthony Isaacs, Ziad Farah

**Affiliations:** 1 Department of Rheumatology, Northwick Park Hospital, London North West University Healthcare NHS Trust, Watford Rd, Harrow, HA1 3UJ UK; 2 Faculty of Medicine, Imperial College London, Exhibition Rd, South Kensington, London, SW7 2BX UK

**Keywords:** Idiopathic inflammatory myopathies, Messenger ribonucleic acid vaccine, Severe acute respiratory syndrome coronavirus 2, Coronavirus disease 2019

## Abstract

**Background:**

Vaccination remains the cornerstone approach to exiting the current global coronavirus disease 2019 pandemic caused by severe acute respiratory syndrome coronavirus 2. The novel messenger ribonucleic acid vaccines offer a high level of protection and are widely used throughout the world. With more people receiving the vaccines, better understanding of their relative safety can be reached. In this report, we describe two patients who developed inflammatory myopathy within 48 hours of receiving the Pfizer BNT162b2 vaccine.

**Case presentation:**

Patient A, a 55-year-old South East Asian woman, presented with a 6-week history of pruritic facial and torso rash and a 1-week history of worsening proximal myopathy. Her rash first developed 2 days after receiving the first dose of BNT162b2 vaccine. Patient B, a 72-year-old Caucasian woman, presented with a 2-week history of proximal myopathy a day after receiving the second dose of BNT162b2 vaccine. Both patients had elevated creatine kinase on admission. Patient A tested positive for anti-Mi-2a antibody and anti-Ro-52 antibody, while Patient B was positive for anti-fibrillarin antibody. Magnetic resonance imaging subsequently confirmed generalized acute muscle inflammation and subcutaneous inflammation consistent with inflammatory myositis. Both patients did not have a previous history or family history of autoimmune disease. Patients A and B were diagnosed with dermatomyositis and inflammatory myositis, respectively. They were initially treated with pulsed intravenous methylprednisolone followed by oral prednisolone. However, as their conditions were resistant to corticosteroids, both eventually received and responded well to intravenous immunoglobulin therapy.

**Conclusion:**

There are previously reported cases of severe acute respiratory syndrome coronavirus 2-induced and other vaccine-related inflammatory myopathies. However, the precise mechanisms are not elucidated. Without more evidence and convincing pathophysiology, it is not possible to conclude that our patients developed inflammatory myopathy because of the vaccine. However, the timing of the disease onset and the lack of previous history raise an important question of this novel messenger ribonucleic acid therapy.

## Background

Coronavirus disease 2019 (COVID-19) is a disease caused by severe acute respiratory syndrome coronavirus 2 (SARS-CoV-2) that was first identified in Wuhan, China, in December 2019. Since then, COVID-19 has become a global pandemic, infecting over 259 million people and causing over 5 million deaths worldwide [1]. Several vaccines against SARS-CoV-2 have been developed and approved, including the novel messenger ribonucleic acid (mRNA) vaccines. Studies have shown that these vaccines are safe, highly effective, and can avert serious symptoms of severe COVID-19, and reduce hospitalizations and mortality [2]. In this case series, we describe two patients who developed idiopathic inflammatory myopathy (IIM) within 48 hours of receiving the BNT162b2 vaccine.

## Case presentation

**Fig. 1:**
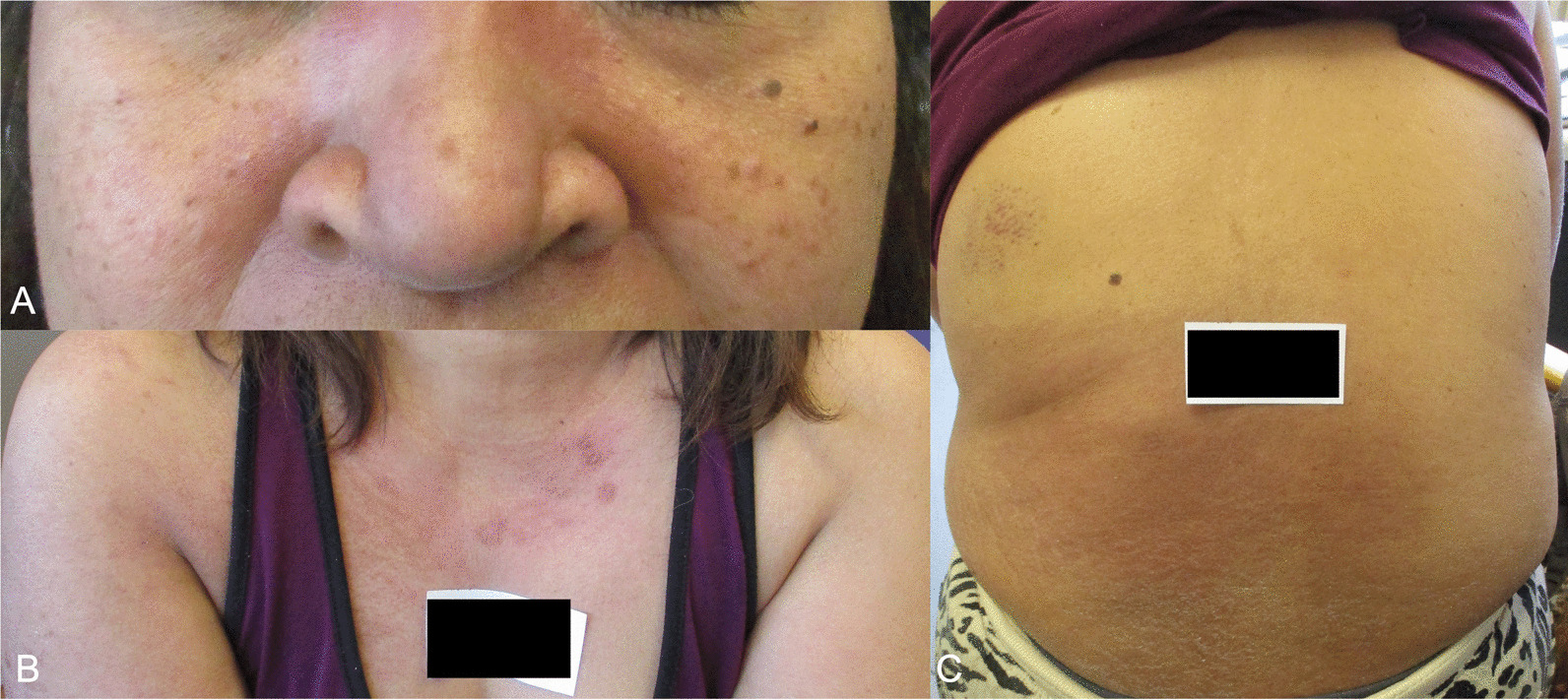
Images of erythematous rash on Patient A seen on the face (**A**), upper torso and lateral aspect of both arms (**B**), and across the lower back (**C**).

Patient A, a 55-year-old South East Asian (Filipino) woman, presented with a 6-week history of pruritic facial and torso rash and a 1-week history of worsening proximal myopathy. Her rash first developed 2 days after receiving the first dose of Pfizer BNT162b2 vaccine. She had a background of type II diabetes mellitus, stage IV chronic kidney disease (CKD), and asthma. There was no family history of autoimmune conditions. She had no drug allergies, regularly took amlodipine, omeprazole, aspirin, sodium bicarbonate, Humalog Mix50, semaglutide, and iron supplements, and used salbutamol inhalers when required. The patient was previously fit and well and worked as a nurse. She lived independently, denied any cigarette smoking and only consumed alcohol occasionally. The proximal power in her shoulders and hips was four on the Medical Research Council (MRC) scale with preserved distal power. Sensation was preserved in all modalities. There was erythematous rash on her face, upper torso, lateral aspects of both arms, and across her lower back (Fig. 1). Her heart sounds were normal and chest was clear. No other significant findings were noted. Vital signs were within normal range.

**Fig. 2:**
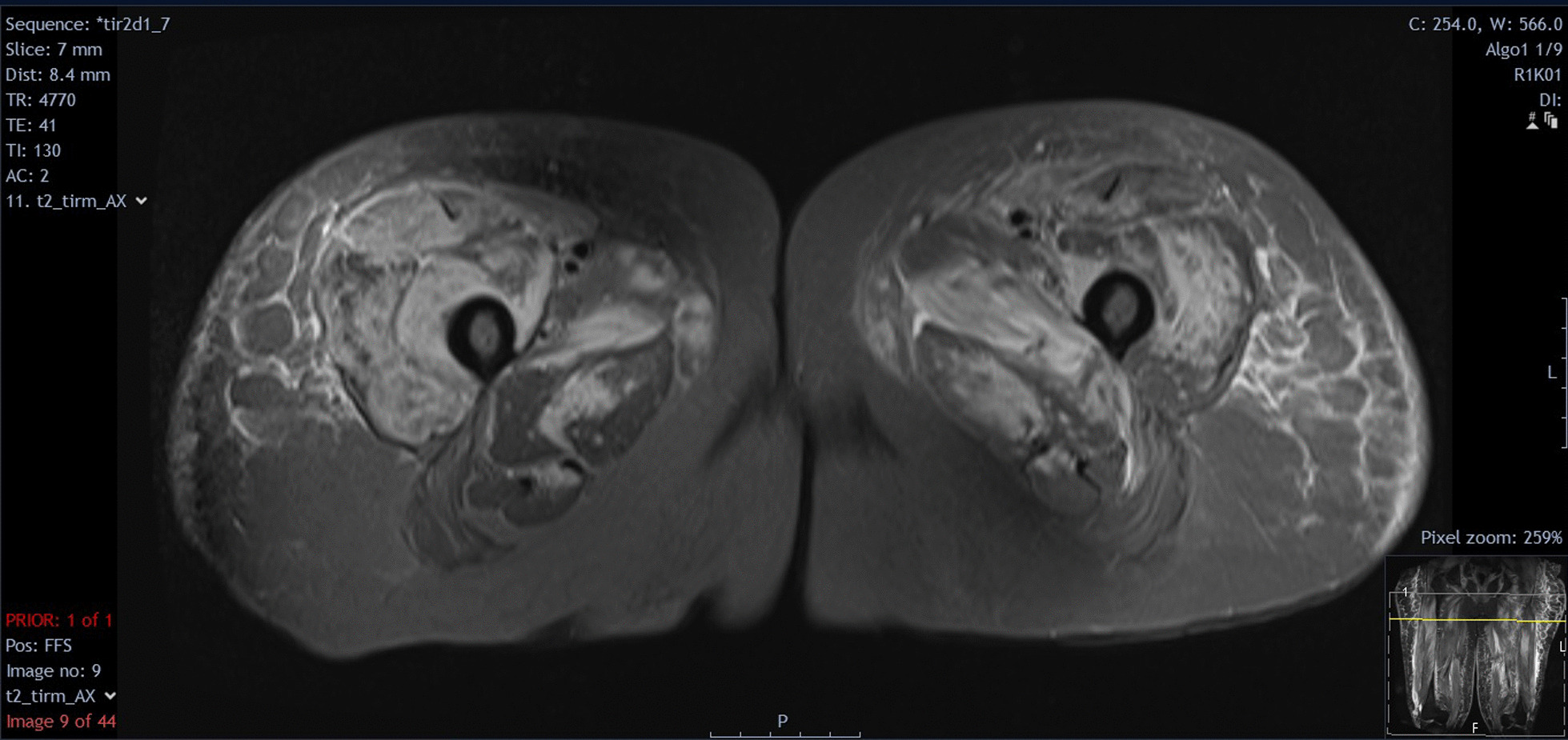
Magnetic resonance imaging T2 with Short Tau Inversion Recovery (STIR) sequences of patient’s proximal thighs showing edema in the vastus lateralis and gluteus muscles more pronounced on the right

Initial blood tests showed a raised creatine kinase (CK) 11330 IU/L, erythrocyte sedimentation rate 111 mm/hr, and C-reactive protein (CRP) 14.1 mg/L. The rest of the initial investigations were as follows: hemoglobin 110 g/L, white cell count 12 × 10^9^/L, neutrophil count 10.2 × 10^9^/L, lymphocyte count 0.7 × 10^9^/L, monocytes 0.6 × 10^9^/L, eosinophils 0.2 × 10^9^/L, platelet count 304 × 10^9^/L, creatinine 170 μmol/L (at baseline), sodium 137 mmol/L, potassium 5.4 mmol/L, corrected calcium 2.07 mg/dL, phosphate 1.6 mmol/L (known CKD), HbA1C 49 mmol/mol, alanine transaminase 132 IU/L, bilirubin 7 μmol/L, alkaline phosphatase 85 IU/L, and albumin 33 g/L. A myositis antibody panel was positive for anti-Mi-2a antibody and anti-Ro-52 antibody. Magnetic resonance imaging (MRI) of lower limbs and pelvis showed edema in the vastus lateralis and gluteus muscles, which were more pronounced on the right. These generalized acute muscle inflammation and subcutaneous inflammation were consistent with inflammatory myositis (Fig. 2). A computed tomography (CT) scan of the chest, abdomen, and pelvis did not reveal any evidence of malignancy.

During admission, the patient developed dyspnoea and chest pain without overt clinical features of cardiac failure. Her troponin T and brain natriuretic peptide peaked at 1684 ng/L and 500 pg/mL, respectively. Electrocardiogram was normal. Echocardiogram showed a preserved left ventricular ejection fraction of 62% and moderate diastolic dysfunction. Cardiac MRI demonstrated nondilated left ventricle with preserved overall function and normal wall thickness and contractility. Short tau inversion recovery images showed no evidence of edema. The patient could not tolerate further imaging with intravenous gadolinium.

She was diagnosed with dermatomyositis and treated with 3 days of pulsed intravenous methylprednisolone followed by oral prednisolone 40 mg once daily. As her condition was resistant to corticosteroids and her symptoms did not improve after 1 week, she received intravenous immunoglobulin (IVIG) and subsequently cyclophosphamide because of clinical concern of cardiac involvement, with good clinical response.

Patient A was followed up by the rheumatology and dermatology teams. Five months after the initial presentation, her proximal myopathy had completely resolved and there were no signs of cardiac failure. She had returned to work. However, she still experienced ongoing rashes on her face and chest, and was started on mycophenolate mofetil maintenance dose while remaining on prednisolone weaning regimen.

Patient B, a 72-year-old White British woman, presented with a 2-week history of proximal myopathy, reduced appetite, painless jaundice, and dark urine. Her symptoms developed a day after receiving the second dose of Pfizer BNT162b2 vaccine. Prior to her acute condition, she did not have any history of muscle weakness. She had a history of out-of-hospital cardiac arrest 7 years ago, and no other medical conditions. Her regular medications included atorvastatin, aspirin, bisoprolol, and omeprazole. There were no drug allergies. She was a retiree who was previously fit and well and lived independently. She denied any cigarette smoking and only consumed alcohol occasionally. On examination, she was unable to get out of bed. The powers on her proximal (shoulders and hips) and distal muscles were two and four on the MRC scale, respectively. Sensation was preserved in all modalities. Patient had normal heart sounds, clear chest, and a normal swallow. No other significant findings were noted. She had a CK of 10,222 IU/L, creatinine of 721 μmol/L, and tested positive for anti-fibrillarin antibody. The rest of the initial blood tests were as follows: hemoglobin 144 g/L, white cell count 10.5 × 10^9^/L, neutrophil count 8.6 × 10^9^/L, lymphocytes 1.4 × 10^9^/L, monocytes 1.0 × 10^9^/L, eosinophils 0.1 × 10^9^/L, platelet count 181 × 10^9^/L, CRP 75.1 mg/L, sodium 135 mmol/L, potassium 3.7 mmol/L, corrected calcium 1.92 mg/dL, phosphate 2.24 mmol/L, parathyroid hormone 38.4 pmol/L, alanine transaminase 246 IU/L, bilirubin 103 μmol/L, alkaline phosphatase 1569 IU/L, and albumin 43 g/L. CT scan showed a suspected pancreatic head tumor with no evidence of metastasis. Endoscopic retrograde cholangiopancreatography revealed a biliary stricture and a biliary brushing cytology showed cells that were suspicious of malignancy.

Prior to imaging, differential diagnoses included rhabdomyolysis secondary to statin and omeprazole usage, and statin-induced necrotizing myositis. However, the patient tested negative for anti-3-hydroxy-3-methylglutaryl coenzyme A reductase antibody and other etiologies were deemed more likely. She was diagnosed with inflammatory myositis secondary to either pancreatic malignancy or SARS-CoV-2 vaccine. Similar to Patient A, she was initially treated with pulsed intravenous methylprednisolone and oral prednisolone 40 mg once daily, and subsequently received IVIG due to progressive myopathy resistant to 6 days of corticosteroids. Her condition improved clinically and biochemically following a gradual taper of corticosteroid therapy. She was later transferred to a hepatobiliary center for management of her malignancy, where a subsequent pancreas biopsy revealed a moderately to poorly differentiated ductal adenocarcinoma with extensive infiltration (T2 N2 Mx R1 staging). She received a Whipple’s procedure and would be followed-up at that center.

Both cases were reported to the Medicines and Healthcare Products Regulatory Agency via the Yellow Card Scheme.

## Discussion and conclusions

IIMs are a group of conditions that include dermatomyositis, polymyositis, and immune-mediated necrotizing myopathy. They share common clinical features such as muscle weakness and inflammation, skin disease, or other organ-specific manifestations. Variation in extramuscular findings, serology, and biopsy results differentiates one condition from another [3].

At present, there is only one reported case of SARS-CoV-2 vaccine-related IIM, where the myositis was localized to the location of intramuscular injection [4]. The patient was managed conservatively without any immunosuppressants. The authors postulated that in addition to the minor muscle injury from injection, there were unknown mechanisms driving the inflammatory process. In our cases, the myositis was systemic and severe, and both patients required and responded well to corticosteroid and immunoglobulin therapy.

Without more cases and convincing pathophysiology, it is not possible to conclude that our patients developed inflammatory myopathy because of the SARS-CoV-2 vaccine, especially in Patient B who might have developed myositis as a paraneoplastic complication of pancreatic malignancy, a well-recognized association [5]. It is also possible that both patients were predisposed to autoimmune disease, whether from preexisting cancer or otherwise, and the vaccine provided the final necessary stimulus to propagate the cascade of autoimmunity to manifest in clinical disease. Regardless, the timing of the disease onset shortly after the vaccine administration and the lack of previous history and family history of autoimmune disorders raise an important question of this novel mRNA therapy.

There are a few proposed mechanisms in drug-induced myopathy: direct myotoxicity, indirect muscle damage, and immunologically related inflammation. Drugs that directly cause myotoxicity, such as glucocorticoids, accumulate in muscle tissues and damage them, while others can indirectly damage tissue by inducing hyperthermia or hyperkinesis [6]. If there is a causal link between mRNA vaccines and IIMs, the process is likely immunological since mRNA and lipid vector are not known to damage muscle tissues directly or indirectly. Cases of immunologically related drug-induced myopathy have been reported in immune checkpoint inhibitors and interferon-alpha, although the precise mechanisms are not elucidated [7].

SARS-CoV-2 infection itself has been associated with autoimmune conditions such as pediatric inflammatory multisystemic syndrome and Guillain–Barré syndrome [8]. Furthermore, a recent study in dermatomyositis patients identifies three T cell receptor epitopes specific to SARS-CoV-2 (O-ribose methyltransferase, RNA-dependent RNA polymerase, and 3′-to-5′ exonuclease proteins), suggesting a potential for the virus to contribute to myositis development [9]. mRNA vaccines, which contain a genetic blueprint for SARS-CoV-2 spike protein, may therefore trigger a similar process. Theoretically, mRNA may bind to pattern recognition receptors prior to translation, be recognized by Toll-like receptors and activate pro-inflammatory cascade including type I interferon response, which is linked to dermatomyositis [10]. In other cases of vaccine-related autoimmune diseases such as the A/New Jersey influenza vaccine and Guillain–Barré syndrome, it is postulated that molecular mimicry or genetic susceptibility are responsible for this association, however, none has been proven [11].

A recent review article discusses various neurological autoimmune disorders encountered within 1–4 weeks of SARS-CoV-2 vaccination [12]. These included demyelinating disease, inflammatory peripheral neuropathies, and two patients with de novo inflammatory myositis. Similar to our two patients here, both cases were female, and both required corticosteroid therapy and improved with immunosuppression. The authors concluded that the incidence of these autoimmune events were low, patient outcomes were favorable, and the benefits of vaccination outweighed the comparatively small risks.

Indeed, in this report, we described two cases of IIMs that developed shortly after receiving the BNT162b2 vaccine. However, without more robust evidence, it is not possible to conclude that there is a causal relationship between the two. Furthermore, even if there was an association between IIM and SARS-CoV-2 vaccine, the risk is extremely small given that there were over 617,000 people who received at least one dose of the vaccine in the boroughs covered by our hospital at the time [13], translating to 1 case in 308,000 individuals. In contrast, SARS-CoV-2 has infected over 170 million people and caused 3.5 million deaths globally [1], highlighting the drastic benefits of the vaccine over its potential harm. Hence, mRNA vaccines against SARS-CoV-2 are widely considered to be safe and effective and should be encouraged to tackle this global pandemic.

## Data Availability

The datasets used during the current study are available from the corresponding author on reasonable request.

## Abbreviations

COVID-19: Coronavirus disease 2019
SARS-Cov-2: Severe acute respiratory syndrome coronavirus 2
mRNA: Messenger ribonucleic acid
IIM: Idiopathic inflammatory myopathy
MRC: Medical Research Council
CK: Creatine kinase
CT: Computed tomography
IVIG: Intravenous immunoglobulin
